# Supplementary material for: Detection of Foodborne Viruses in Dates Using ISO 15216 Methodology
Source: Viruses. 2025 Jan 26;17(2):174. doi: 10.3390/v17020174 (PMC11860475; doi:10.3390/v17020174)
Supplement: Supplementary file 1 [file viruses-17-00174-s001.zip › viruses-3405876-supplementary.pdf]

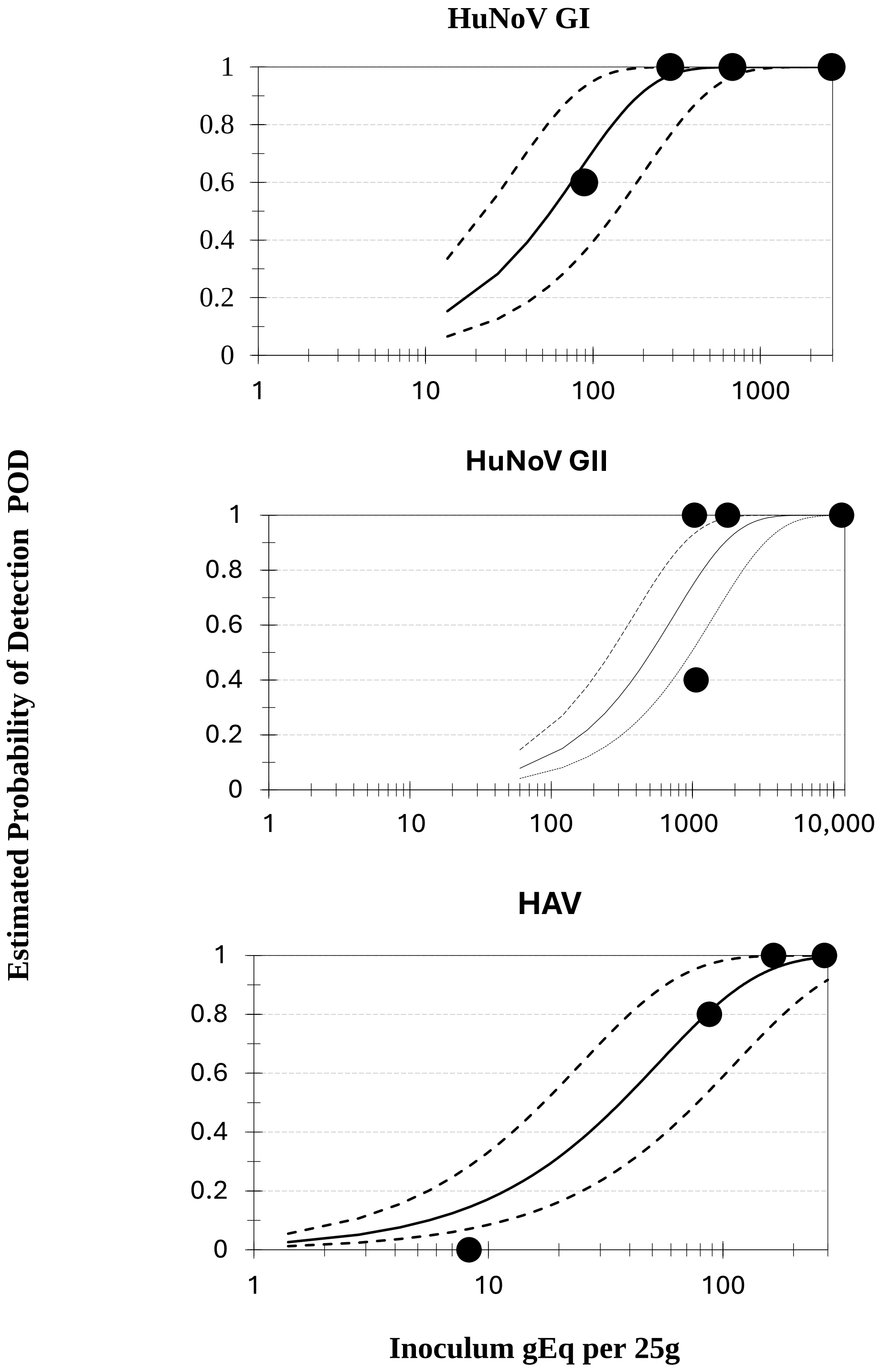

Supplementary Figure S1: **Estimated probability of detection (POD) curve of MNV, HuNoV GI, HuNoV GII and HAV extracted from pitted dates using ISO-modC.** The estimated POD curve for the viruses spiked on pitted dates (P-Uns/Iran/M) detected by RT-qPCR (solid lines) and the 95% confidence interval for the POD (dashed lines). Each observed value represents the ratio of positive results from three to five extractions tested using RT-qPCR (●).

Supplementary Table S1. List of primers, probes, and RNA transcripts[*1]

Target virus	Primer	5'-3' Sequence	Reference
HAV	F:SH-Poly-A	GARTTTACTCAGTGTTCAATGAATGT	40
	R:SH-Poly-1	GGCATAGCTGCAGGAAAATT	40
	P:SH-Poly-Q	FAM–TCTCCAAAA(ZEN)CGCTTTTTAGAAAGAGTCC–3IABkFQ	41
	RNA transcript	GARTTTACTCAGTGTTCAATGAATGTAGTCTCCAAAACGCTTTTTAGAAAGAGTCCCATTTATCATCACATTGATAAAACCATGAT*ACTGAGGGTTGCGTTAGACGGGCGACAGAT*TAATTTTCCTGCAGCTATGCC	26
HuNoV GI	F:QNIF4	CGCTGGATGCGNTTCCAT	42
	R:NV1LCR	CCTTAGACGCCATCATCATTTAC	43
	P:TM9-MGB	FAM–TGGACAGGAGATCGC–MGB–NFQ	44
	RNA transcript	GGGCGAATTGGGTACGATCGATGCGGCCTCGATATCCGCTGGATGCGCTTCCATGACCTCGGATTGTGGACAGGAGATCGCGATCTTCTGCCC*ACTGAGGGTTGCGTTAGACGGGCGACAGATCGT*CGAATTCGTAAATGATGATGGCGTCTAAGGA	28
HuNoV GII	F:QNIF2	ATGTTCAGRTGGATGAGRTTCTCWGA	45
	R:COG2R	TCGACGCCATCTTCATTCACA	46
	P:QNIFS	6FAM–AGCACGTGGGAGGGCGATCG–BHQ1	45
	RNA transcript	GGGCGAATTGGGTACGATCGATGCGGCCTCGAATTCATGTTCAGATGGATGAGATTCTCAGATCTGAGCACGTGGGAGGGCGATCGCAATCTGGCTCCCAGT*ACTGAGGGTTG*TTTGTGAATGAAGATGGCGTCGAA	28
MNV	F:Q-MNV-1dR	CACGCCACCGATCTGTTCTG	47
	R:Q-MNV-1dF	GCGCTGCGCCATCACTC	47
	P:Q-MNV-1d-MGB	FAM–CGCTTTGGAACAATG–MGBNFQ	47
	RNA transcript	CACGCCACCGATCTGTTCTGCGCTGGGTGCGCTTTGGA<br>ACAATGGATGCTGAGACC*ACTGAGGGTTGCGTTAGA*CGGCCGCAGG<br>AACGCTCAGCAGTCTTTGTGAATGAGGATGAGTGATGGCGCAGCGC	48

[*1] The RNA transcript inserts are underlined and italicized

Supplementary Table S2. MNV recovery and contamination assays from pitted and whole dates.

Sample	Analyst	HAV Ct	HuNoV GI Ct	HuNoV GII Ct	MNV Recovery (%)
P-Uns/USA-Algeria/I-301	1	nd	nd	nd	4.3
P-Uns/USA-Algeria/I-302	1	nd	nd	nd	1.6
P-Uns/Algeria/I-409	2	nd	nd	nd	1.2
P-Deglet noor/Tunisia-Algeria/J1-311	1	nd	nd	nd	2.7
P-Deglet noor/Tunisia-Algeria/J1-312	1	nd	nd	nd	1.4
P-Deglet noor/Tunisia-Algeria/J1-413	2	nd	nd	nd	0.8
P-Deglet noor/Tunisia-Algeria/J2-349	1	nd	nd	nd	2.0
P-Deglet noor/Tunisia-Algeria/J2-350	1	nd	nd	nd	4.3
P-Deglet noor/Tunisia-Algeria/J2-414	2	nd	nd	nd	1.2
P-Medjool/USA/A1-351	1	nd	nd	nd	36.7
P-Medjool/USA/A1-352	1	nd	nd	nd	64.5
P-Medjool/USA/A1-415	2	nd	nd	nd	10.0
P-Medjool/USA/A2-303	1	nd	nd	nd	20.0
P-Medjool/USA/A2-304	1	nd	nd	nd	14.0
P-Medjool/USA/A2-410	2	nd	nd	nd	1.9
W-Medjool/USA/K-305	1	nd	nd	nd	13.7
W-Medjool/USA/K-306	1	nd	nd	nd	28.8
W-Medjool/USA/K-411	2	nd	nd	nd	3.8

P-Uns/Unk/L-307	1	nd	nd	**41.9**	7.2
P-Uns/Unk/L-308	1	nd	**39.2**	nd	13.3
P-Uns/Unk/L-412	2	nd	nd	**41.7**	10.0
P-Uns/Iran/M1-309	1	nd	nd	nd	4.9
P-Uns/Iran/M1-310	1	nd	nd	nd	8.7
P-Uns/Iran/M2-359	1	nd	**40.8**	nd	36.7
P-Uns/Iran/M2-360	1	nd	nd	nd	12.7
P-Uns/Iran/M2-419	2	nd	nd	nd	28.3
P-Uns/Algeria/N-353	1	nd	nd	nd	4.0
P-Uns/Algeria/N-354	1	nd	nd	nd	1.2
P-Uns/Algeria/N-416	2	nd	nd	nd	0.7
P-Uns/Iran/O-355	1	nd	nd	nd	26.5
P-Uns/Iran/O-356	1	nd	nd	nd	29.0
P-Uns/Iran/O-417	2	nd	nd	nd	5.1
P-Uns/Palestine/P-357	1	nd	nd	nd	27.9
P-Uns/Palestine/P-358	1	nd	**40.1**	nd	61.8
P-Uns/Palestine/P-418	2	nd	**42.2**	nd	13.9

Supplementary Table S3. RT-qPCR inhibition rates from pitted and whole dates.

Varieties	EAC inhibition[*1]			Inhibition 1/10 dilution[*2]
	HuNoV GI	HuNoV GI	HAV	MNV
P-Uns/USA-Algeria/I	-15%	1%	26%	19%
P-Medjool/USA/A1	-6%	-2%	4%	42%
W-Medjool/USA/K	-18%	1%	12%	14%
P-Uns/UnK/L	10%	2%	29%	14%
P-Deglet noor/Tunisia-Algeria/J1	-26%	-57%	-13%	nd
P-Deglet noor/Tunisia-Algeria/J2	-13%	-33%	-9%	56%
P-Medjool/USA/A1	-22%	-23%	-21%	7%
P-Uns/Algeria/N	-22%	-55%	9%	36%
P-Uns/Iran/O	-14%	-17%	4%	-25%
P-Uns/Palestine/P	-4%	-12%	22%	1%
P-Uns/Iran/M2	-9%	-9%	18%	8%

n=1

nd= not determined, the diluted RNA extract was not detected.

[*1] EAC RT-qPCR inhibition was calculated using the formula = ($10^{(\Delta Ct/m)}$ × 100%) where ΔCt is the Ct value of viral RNA extract from the matrix minus the Ct value of the inoculum, and m is the slope of the virus RNA transcript standard curve.

[*2] The MNV RT-qPCR inhibition was estimated using the ratio of diluted 1/10 MNV RNA extracted. The RT-qPCR inhibition = ($1-10^{(\Delta Ctdil/m-1)}$) × 100% where ΔCtdil is the Ct value of viral RNA extract from the matrix diluted 1/10 in RNase free water minus the Ct value of the undiluted RNA extracted, and m is the slope of the virus RNA transcript standard curve.

References

26. Larocque, E.; Levesque, V.; Lambert, D. Crystal digital RT-PCR for the detection and quantification of norovirus and hepatitis A virus RNA in frozen raspberries. *Int. J. Food Microbiol.* **2022**, *380*, 109884.
28. Raymond, P.; Paul, S.; Perron, A.; Bellehumeur, C.; Larocque, E.; Charest, H. Detection and Sequencing of Multiple Human Norovirus Genotypes from Imported Frozen Raspberries Linked to Outbreaks in the Province of Quebec, Canada, in 2017. *Food Environ. Virol.* **2022**, *14*, 40–58.
40. Guevremont, E.; Brassard, J.; Houde, A.; Simard, C.; Trottier, Y.L. Development of an extraction and concentration procedure and comparison of RT-PCR primer systems for the detection of hepatitis A virus and norovirus GII in green onions. *J Virol Methods* **2006**, *134*, 130-5.
41. Houde, A.; Guevremont, E.; Poitras, E.; Leblanc, D.; Ward, P.; Simard, C.; Trottier, Y.L. Comparative evaluation of new TaqMan real-time assays for the detection of hepatitis A virus. *J Virol Methods* **2007**, *140*, 80-9.
42. da Silva, A.K.; Le Saux, J.C.; Parnaudeau, S.; Pommepuy, M.; Elimelech, M.; Le Guyader, F.S. Evaluation of removal of noroviruses during wastewater treatment, using real-time reverse transcription-PCR: different behaviors of genogroups I and II. *Appl Environ Microbiol* **2007**, *73*, 7891-7.
43. Svraka, S.; Duizer, E.; Vennema, H.; de Bruin, E.; van der Veer, B.; Dorresteijn, B.; Koopmans, M. Etiological role of viruses in outbreaks of acute gastroenteritis in The Netherlands from 1994 through 2005. *J Clin Microbiol* **2007**, 45, 1389-94.
44. Hoehne, M.; Schreier, E. Detection of Norovirus genogroup I and II by multiplex real-time RT- PCR using a 3'-minor groove binder-DNA probe. *BMC Infect Dis* **2006**, *6*, 69.
45. Loisy, F.; Atmar, R.L.; Guillon, P.; Le Cann, P.; Pommepuy, M.; Le Guyader, F.S. Real-time RT-PCR for norovirus screening in shellfish. *J Virol Methods* **2005**, *123*, 1-7.
46. Kageyama, T.; Kojima, S.; Shinohara, M.; Uchida, K.; Fukushi, S.; Hoshino, F.B.; Takeda, N.; Katayama, K. Broadly reactive and highly sensitive assay for Norwalk-like viruses based on real-time quantitative reverse transcription-PCR. *J Clin Microbiol* **2003**, *41*, 1548-57.
47. Baert, L.; Wobus, C.E.; Van Coillie, E.; Thackray, L.B.; Debevere, J.; Uyttendaele, M. Detection of murine norovirus 1 by using plaque assay, transfection assay, and real-time reverse transcription-PCR before and after heat exposure. *Appl Environ Microbiol* **2008**, *74*, 543-6.
48. Raymond, P.; Paul, S.; Guy, R.A. Impact of Capsid and Genomic Integrity Tests on Norovirus Extraction Recovery Rates. Foods **2023**, *12*, 826.
